# La “nueva odontología” en tiempos de la “nueva normalidad”

**DOI:** 10.21142/2523-2754-0901-2021-052

**Published:** 2021-03-11

**Authors:** Guido Alberto Perona-Miguel de Priego, Sabina Mungi Castañeda

**Affiliations:** 1 Universidad Peruana Cayetano Heredia. Lima, Perú. guido.perona@upch.pe Universidad Peruana Cayetano Heredia Universidad Peruana Cayetano Heredia Lima Peru guido.perona@upch.pe; 2 Universidad Científica del Sur. Lima, Perú. smungic@ucientifica.edu.pe Universidad Científica del Sur Universidad Científica del Sur Lima Peru smungic@ucientifica.edu.pe

**Keywords:** COVID-19, coronavirus, atención odontológica, bioseguridad, COVID-19, Coronavirus, Dental Care, Biosecurity

## Abstract

El propósito de este trabajo es proporcionar a los cirujanos dentistas que atienden a niños y adolescentes, durante y después de la pandemia de COVID-19, sugerencias de atención con alternativas de tratamiento mínimamente invasivas para disminuir el riesgo de infección viral cruzada y ofrecer un entorno clínico más seguro.

## Abstract (en)

The aim of this study was to provide health professionals, especially to dental surgeons attending children and adolescents, care guidelines recommending minimally invasive treatment alternatives to minimize the risk of cross viral infection and providing a safer clinical environment during and after the COVID-19 pandemic.

## INTRODUCCIÓN

Mientras celebrábamos las fiestas de fin del año 2019 en el Perú, en China (Wuhan) se presentaron casos de neumonía sin causa determinada, acompañados de aumento de temperatura, cansancio, tos y disnea de aparición rápida. Se identificó una nueva versión de coronavirus, lo que fue confirmado luego por la Organización Mundial de la Salud (OMS). Este virus fue denominado virus 2019-nCoV y luego, SARS-CoV-2; mientras que a la neumonía se la nombró como COVID-19 ^1^. Esta familia de virus es zoonótica, lo que significa que se transmite de animales a humanos. Otros miembros de ella son el coronavirus del síndrome respiratorio agudo severo (SARS-CoV) y el coronavirus del síndrome respiratorio del Medio Oriente (MERS-CoV), encontrados en 2002 y 2012, respectivamente. ^2^

El 30 de enero de 2020, la OMS (Organización Mundial de la Salud) declaró que la infección por COVID-19 era una emergencia de salud pública y el 11 de marzo pasó de ser una epidemia a una pandemia. Según la OMS (al 13 de julio de 2020), se han notificado más de 12,945,505 millones de casos a nivel mundial, que afectan a más de 170 países y este número continúa aumentando ^3^.

El virus comenzó a manifestarse en diversos países de Asia, Europa y llegó a Latinoamérica. El Perú no fue la excepción y el primer caso (paciente 0) confirmado se conoció el viernes 6 de marzo del 2020. Se trató de un individuo de 25 años, trabajador de una aerolínea comercial que había estado de vacaciones en Europa, donde contrajo el virus, y que manifestó los síntomas a su llegada al país. Luego de un confinamiento, se recuperó totalmente de la enfermedad, pero contagió a su entorno de familiares y amigos, lo cual demostró el grado de agresividad de este virus. El 19 de marzo se registró el primer fallecido en el Perú , un varón de 78 años internado en el Hospital Central de la Fuerza Aérea del Perú ^4^.

El contagio se produce de persona a persona, en forma directa o indirecta, por proximidad, y se propaga a través de la saliva y los fluidos respiratorios ^(4, 5)^. Las personas afectadas presentan síntomas clínicos típicos como malestar general, cefalea, tos seca, fiebre, mialgia o fatiga, dificultad para respirar, diarrea, conjuntivitis, inflamación de dedos en ambas extremidades ^(6, 7)^, así como pérdida parcial o total del olfato (hiposmia/anosmia) y cambio o pérdida del sentido del gusto. Si se complica el cuadro, el paciente puede desarrollar una neumonía grave con insuficiencia orgánica múltiple ^8^. Se viene observando que las personas mayores y las que padecen afecciones médicas previas, como hipertensión arterial, problemas cardiacos o pulmonares, diabetes o cáncer, tienen más probabilidades de presentar cuadros graves ^9^.

Por lo general, las personas entre 25 y 89 años se ven afectadas, con una ligera prevalencia en los hombres; sin embargo, no se puede generalizar. Se ha informado una menor incidencia en niños, lo que podría deberse a que los receptores ACE 2 (enzima convertidora de angiotensina 2) se encuentran inmaduros, la presencia de anticuerpos contra diferentes virus y un sistema inmune en desarrollo que reacciona de manera diferente al virus, según lo postulado por Dong et al. Los niños presentan síntomas similares a los adultos, pero en forma más leve; debido al prolongado período de incubación, los niños pueden tener una fase asintomática o presentar síntomas leves y no específicos. En consecuencia, todos los niños deben ser considerados portadores potenciales de la COVID-19, a menos que se demuestre lo contrario ^10^.

Los modos de transmisión incluyen ^1^ la transmisión directa o aerosol (por tos, estornudos e inhalación de gotas) y ^2^ la transmisión por contacto (a través de la mucosa nasal, oral y ocular). Se considera el modo de transmisión más frecuente las gotas de saliva o fluidos (> 5-10 µ m) cargadas con el virus, desde una aproximación cercana menor de 2 metros. Existe un alto riesgo de que la mucosa oral de la persona infectada tenga un recuento alto del virus. La transmisión también se produce a través de fómites presentes en las inmediaciones de la persona infectada. Como resultado, el virus puede permanecer vivo en las superficies con las que tuvo contacto la persona infectada durante un período mínimo de 9 días ^11^.

El personal de salud: médicos, enfermeras, auxiliares de enfermería y técnicos presentan un alto riesgo de contagio por el contacto y la atención de los pacientes afectados, así como el personal administrativo que tiene contacto con estos pacientes.

Los odontólogos tienen un gran riesgo por los diversos procedimientos que involucran aerosoles y por estar en contacto muy cercano con los pacientes, sobre todo con la cavidad bucal, por la que se eliminan sangre, saliva y microorganismos ^13^. Se ha demostrado que la mucosa oral tiene una importante relación con la infección de COVID-19, además de expresar el receptor ACE2 en las glándulas salivales durante el proceso asintomático, por lo que la saliva infectada es una de las principales fuentes de virus ^12^.

Asimismo, las superficies contaminadas en el lugar de trabajo perpetúan la supervivencia del virus hasta por cinco días ^(12, 13)^. A pesar de la posible transmisión y contaminación, todavía no hay estudios que evalúen la magnitud de transmisión del SARS-CoV-2 durante el cuidado dental o determinen la descripción del riesgo para el equipo dental con respecto a la exposición y el riesgo de contaminación cruzada ^13^. Todas las recomendaciones que se emitan deben estar dirigidas a prevenir y minimizar el riesgo potencial tanto para profesionales como para pacientes y cuidadores, durante y después de la pandemia de coronavirus ^16^.

Varias organizaciones dentales han declarado que solo se realizarán procedimientos dentales de emergencia y la odontología pediátrica no es la excepción. La Asociación Americana de Odontología Pediátrica (AAPD) ha aconsejado a los dentistas pediátricos que pospongan todos los procedimientos electivos, pero continúen la atención de emergencia o urgencia. También han sugerido posponer los casos de anestesia general ^10^.

## PROTOCOLOS CLÍNICOS RECOMENDADOS

Diferentes organismos de salud han publicado sugerencias de protocolos para la atención odontológica (Tabla 1). Por ejemplo, la Asociación Latinoamericana de Odontopediatría (ALOP) menciona dos protocolos que se deben seguir: la teleodontología y la atención presencial sea de urgencia o de emergencia. Amorin et al. ^15^ la dividen en cuatro: evaluación del paciente, preoperatorio, quirúrgico y posoperatorio.

**Tabla 1:** Diferentes protocolos emitidos por organismos de salud. Tomado y adaptado de Amorim et al. ^19^

Institutional Agency Document Reference	Institutional Agency Document Reference
World Health Organization (WHO)	Infection prevention and control during health care when novel coronavirus (nCoV) infection is suspected
World Dental Federation (FDI) COVID-19	Outbreak: Guidance for Oral Health Professionals
American Dental Association (ADA)	Return to Work Interim: Guidance Toolkit
Latin American Association of Pediatric Dentistry (ALOP)	Tratamiento de la enfermedad de caries en época de COVID-19: protocolos clínicos para el control de aerosoles
American Academy of Pediatric Dentistry (AAPD)	Re-emergence pediatric dentistry practice checklist
International Association of Paediatric Dentistry (IAPD)	International pulmonologist’s consensus on COVID-19
Ministerio de salud del Perú (Minsa) ^17^	Directiva sanitaria N.° 100 /MINSA/2020/DGIESP-Manejo de la atención estomatológica en el contexto de la pandemia COVID-19

La recomendación general es utilizar primero la teleodontología y tratar de solucionar los casos en lo posible dando orientación a los pacientes, utilizando la comunicación por teléfono o WhatsApp, apoyada por fotos que pudiera tomar el paciente, a fin de evitar los tratamientos electivos presenciales, y priorizar procedimientos urgentes como dolor, edema, hemorragia y trauma dentoalveolar.

**Figura 1:**
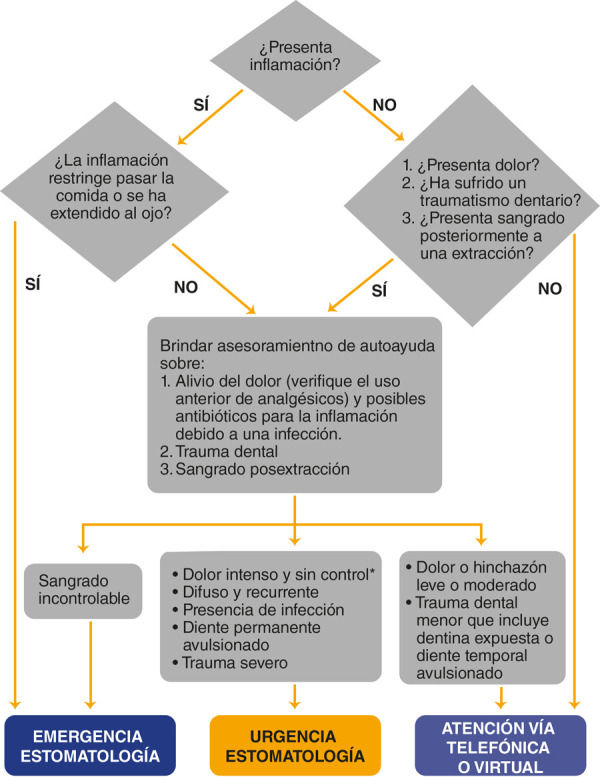
Flujograma para determinar el tipo de atención estomatológica (Minsa, Perú) ^17^

Por un lado, todo odontólogo debe asumir que cualquier paciente puede ser asintomático, ligeramente sintomático o estar en el período de incubación (estimado entre 1 y 14 días), es decir, todos los pacientes deben ser tratados como si estuvieran contaminados ^16^. Es mejor si el paciente puede llevar a la consulta presencial el resultado de una prueba molecular negativa. Por otro lado, el odontólogo y su equipo de auxiliares deben también estar controlados con pruebas, ya que el paciente también tiene el derecho de saber que quien lo trata no está contaminado. Para ello, deben establecerse protocolos internos entre el personal del consultorio previos a la atención, como la toma de temperatura antes de ingresar al consultorio, lavarse las manos con jabón, cambiar la vestimenta de calle por ropa de uso interno, el uso de EPP para todos, mascarillas seguras, desinfección del ambiente entre paciente y paciente, y cambio de material e instrumental (Figura 1).

## PROCESO PARA LA ATENCIÓN DEL PACIENTE EN LA CONSULTA ODONTOLÓGICA EN TIEMPO DE PANDEMIA (ATENCIÓN NO PRESENCIAL)

### 1. Teleodontología

Debemos realizar primero la teleodontología, que es la atención a distancia que involucra el uso de telefonía, mensajes de texto, WhatsApp u otros medios digitales o plataformas virtuales, como Zoom, Teams, Google Meet, Hangouts, Skype, Facebook y Messenger. Se trata de utilizar la tecnología disponible para realizar el diagnóstico, brindar orientación, llevar a cabo el seguimiento y definir quienes requieren atención presencial ^18^.

Las teleodontología se pueden dividir en tres tipos:

- a. Consulta asincrónica, que es aquella en la que el padre del niño contacta al odontopediatra para solicitar una opinión o evaluación. En este caso existe un tiempo de espera entre el envío de la solicitud o requerimiento, y la respuesta.
- b. Consulta sincrónica, cuando la consulta virtual se produce en tiempo real por teléfono o, mejor, por videoconferencia, en la que se puede interactuar con el odontopediatra sobre los motivos de la consulta.
- c. Consulta mixta, cuando se realiza el seguimiento en el mediano o largo plazo de un caso específico, utilizando consultas asincrónicas y sincrónicas ^(19, 20)^.

En cualquiera de estos casos, el odontólogo debe crear un archivo de las comunicaciones, videos y fotos para el seguimiento del caso.

La importancia de la teleodontología radica en que permite, mediante un triaje virtual, determinar si es posible realizar el tratamiento sintomático a distancia o si se requiere un tratamiento presencial, como urgencia o emergencia. Generalmente, la consulta virtual de urgencia se realiza de forma sincrónica. Mostramos los cuadros de urgencia y emergencia odontológica sugeridos por la ADA (American Dental Association) y el Minsa ^17^ (Tabla 2).

**Tabla 2:** Emergencias y urgencias estomatológicas (a, b, c)

a. EMERGENCIA ESTOMATOLÓGICA es toda circunstancia que pone potencialmente en riesgo la vida de las personas y requiere tratamiento o intervención inmediata para detener el sangrado tisular continuo, aliviar dolor intenso o tratar una infección.
Se incluye: • Presencia de sangrado no controlable. • Celulitis o infección difusa en tejidos blandos con manifestación de aumento de volumen intraoral o extraoral, y que compromete potencialmente la vía aérea del paciente y puede requerir drenaje quirúrgico. • Traumatismo que compromete los huesos maxilares y faciales, que potencialmente pueden comprometer la vía aérea del paciente
b. URGENCIA ESTOMATOLÓGICA es toda atención que se enfoca en el manejo de afecciones que requieren atención inmediata para aliviar el dolor intenso o el riesgo de infección, y de esa forma contribuir a aliviar la carga que se pueda presentar en las emergencias hospitalarias. Debe ser tratada de manera mínimamente invasiva, en cuanto sea posible.
Se incluye: • Odontalgia severa por inflamación pulpar. • Pericoronitis o dolor en el tercer molar • Osteítis posquirúrgica • Absceso localizado acompañado de dolor e hinchazón. • Fractura dental con dolor o trauma en los tejidos blandos. Avulsión/luxación dental. • Tratamiento dental requerido antes de procedimientos médicos críticos. • Cementación de la corona/puente, pérdida de restauración temporal, fractura que causa irritación gingival. • Biopsia de tejido anormal. • Caries dental extensa o defectuosa. • Restauraciones desbordantes o fracturadas. • Aplicación de técnicas de restauración provisional cuando sea posible (fluoruro de amino de plata, ionómero de vidrio y otros). • Retiro de suturas. • Ajuste de prótesis dentales en pacientes que reciban tratamiento oncológico. • Ajustes o reparaciones de prótesis dentales cuando la función masticatoria está impedida o limitada. • Lavado de conductos y reemplazo de medicación. • Alambres de ortodoncia sueltos que dañan la mucosa oral.
c. Procedimientos dentales de rutina o no urgentes
Se incluyen, entre otros: • Exámenes orales y visitas iniciales o periódicas, incluidas radiografías de rutina. • Profilaxis de rutina y terapias preventivas. • Controles y procedimientos de ortodoncia mínimos. • Exodoncia tracción de piezas asintomáticos. • Odontología restaurativa y estética.

## PROCESO PARA LA ATENCIÓN DEL PACIENTE EN LA CONSULTA ODONTOLÓGICA EN TIEMPO DE PANDEMIA (ATENCIÓN PRESENCIAL)

Si se determina la atención presencial del paciente, se deben seguir estos pasos:

### 1. Programación

• Toda programación de un paciente debe hacerse con anticipación por medios en línea.
• El paciente debe informar con anticipación si ha tenido fiebre o malestar general las últimas 48 horas. De ser así, debe postergarse la cita.
• El paciente debe llegar con anticipación y usando una mascarilla de protección.
• Se deben limpiar y desinfectar todos los ambientes donde ha permanecido el paciente.
• El paciente debe enviar con anticipación las respuestas a un cuestionario de salud y su consentimiento informado.
• Si el paciente tiene historia de positivo para COVID-19 y necesita asistencia presencial, debe ser citado al final del día.
• Si el paciente es un niño, se debe acondicionar su conducta con videos sobre el entorno del consultorio, la vestimenta del personal y las medidas de protección que se le brindará tanto a él como a su acompañante, si se requiere su presencia.

### 2. Periodo preoperatorio

• El consultorio, desde su ingreso y en lugares estratégicos, debe mostrar alertas visuales (letreros y carteles) como recursos alternativos para reforzar las instrucciones de bioseguridad.
• La sala de espera debe tener espacio entre las sillas. Todos los objetos comunes de manipulación, como revistas y juguetes, deben ser retirados para evitar superficies expuestas a la contaminación.
• Debe haber disponibilidad y fácil acceso a alcohol en gel para manos en diferentes ambientes.
• Todas las ventanas de los ambientes deben estar abiertas para que circule el aire.
• Si se usa aire acondicionado, deberá tener filtros de alto rendimiento.
• Al llegar el paciente y su acompañante, el personal auxiliar debe tomar la temperatura corporal con un termómetro infrarrojo y evitar saludos de mano (si hay fiebre, es preciso postergar la cita).
• Entregar y ayudar al paciente a colocarse su EPP completo: mandilón, zapatos y gorro descartable (igual si lo requiere el acompañante)
• Cuando esté vestido adecuadamente, el paciente se lavará las manos antes de ingresar a la sala de atención y se recomienda el uso de mascarilla, exclusiva y personal, hasta el momento de la propia atención.
• Se recomienda un enjuague bucal con peróxido de hidrógeno al 1%, debido a su potencial oxidativo y la consiguiente reducción de la carga viral de COVID-19. El digluconato de clorhexidina al 0,12%, a su vez, aún carece de evidencia científica más sólida.
• El uso de enjuagues bucales es exclusivamente para el procedimiento previo y no se recomienda el uso continuo por parte del paciente ^21^.

### 3. Periodo operativo

• Lo primero es minimizar los objetos en las superficies, tratar de reducir la contaminación cruzada, quedando solo instrumentos y material de consumo individualizado disponible para que el paciente sea atendido.
• El profesional debe quitarse los aretes, anillos, collares, pulseras y relojes. Los hombres deben evitar el uso de barba y las mujeres, el maquillaje excesivo, para un mejor sellado y efectividad de la mascarilla.
• Con respecto al EPP, el dentista debe usar un respirador tipo N95 o, al menos, PFF-2 (sin válvula); bata impermeable y desechable; gorra; gafas protectoras; guantes desechables y mascarilla ^24^.
• La secuencia correcta del apósito de EPP implica lo siguiente: 1) Ponerse la máscara; 2) Ponerse gafas protectoras; 3) Ponerse la gorra; 4) Ponerse el protector facial; 5) Ponerse la bata desechable; y 6) Ponerse guantes protectores. La eliminación del EPP debe seguir el orden inverso (36).
• Postergar el uso de la pieza de mano de alta velocidad por el aerosol; en cambio, utilizar una pieza de baja velocidad o solo instrumentos manuales, como curetas para dentina.
• Si es inevitable el uso de la alta velocidad, usar aislamiento absoluto, succión de alta potencia y tomar el menor tiempo posible.
• Utilizar procedimientos de odontología mínimamente invasivos, materiales como fluoruro amino de plata, cementos de ionómeros de vidrio y técnica de Hall, hasta que llegue el momento adecuado para realizar tratamientos convencionales.

### 4. Periodo posoperatorio

• Debemos tomar la atención del control y mantenimiento del tratamiento realizado y supervisarlo, sobre todo si el paciente este implicó dolor, sangrado, control de medicación y alivio o complicación.
• En presencia de síntomas sistémicos (fiebre, pérdida de apetito, postración) seguidos de edema y signos infecciosos agudos, la prescripción habitual de antibióticos (suspensión oral o tabletas) se mantiene bajo supervisión estricta.
• Debemos dar una gran importancia a las medidas preventivas como el cepillado dental con pasta con flúor (mínimo 1000 ppm de fluoruro) y uso de hilo dental.
• Después de que el paciente se retira, se deben activar todos los procedimientos de limpieza del ambiente y las superficies. El próximo paciente solo podrá ser atendido después de dos horas.
• Las desinfecciones de los equipos odontológicos deben tener un orden: desde el área menos contaminada hasta la más contaminada; siempre de arriba hacia abajo y desde la parte interna hacia la externa.
• Se recomienda limpiar las superficies con detergente neutro, seguido por la desinfección con soluciones desinfectantes, como alcohol (70%) o hipoclorito de sodio (1%) ^22^.
• El manejo de los residuos debe llevarse a cabo con mucho cuidado, en bolsas impermeables que se colocarán en un contenedor especial para su recojo por un servicio especializado.

Puede ser esencial en este contexto trabajar en el análisis de la situación a través del FODA (fortalezas, oportunidades, debilidades y amenazas), ya que esto nos ayudará a saber qué realidad se enfrenta. También nos ayuda a maximizar nuestra fuerza y minimizar los riesgos asociados. Como resultado, podemos llegar a un plan estratégico para manejar el escenario actual de pandemia ^11^.

## FORTALEZAS

Después de la pandemia, aumentará la demanda de servicios dentales, particularmente la atención de emergencia, por lo que los servicios de salud ocuparán un lugar central en las políticas públicas. 

Las oportunidades pueden aumentar aún más, pues habrá una mayor conciencia en la población general. Por lo tanto, la preparación para brindar estos servicios a los pacientes puede ser la mayor fortaleza de cualquier clínica dental ^11^.

## OPORTUNIDADES

El mantenimiento del enfoque de salud hacia la prevención de enfermedades se convertiría en la necesidad principal. 

El manejo de la pandemia actual también exige innovaciones en las prácticas dentales de rutina, por lo tanto, brinda oportunidades para mejorar los procesos. 

El futuro puede ver una nueva era de investigación y resultados prometedores. Esto ampliaría el alcance de la imaginación y también incorporaría los avances tecnológicos ^11^.

## DEBILIDADES

Realizar cualquier atención de emergencia requeriría el uso de EPP quirúrgico.

El costo y la demanda de EPP aumentarán y deberán ser considerados como inversiones adicionales por parte de los dentistas; este factor se duplica, ya que es necesario realizar estas inversiones no solo para el operador, sino también para el equipo de asistentes dentales.

Aunque puede haber un aumento repentino en la necesidad de servicios dentales, los pacientes inicialmente pueden centrarse más en los servicios de emergencia.

Las capacidades de pago del público también pueden verse afectadas en cierta medida.

Puede haber dificultades para adquirir suministros dentales, lo que implicará la necesidad de modificaciones en los protocolos de tratamiento dental ^11^.

## AMENAZAS

La falta de evidencia científica sobre el curso de la enfermedad puede hacer que los protocolos de salud cambien constantemente.

Los organismos estatutarios superiores y los encargados de formular políticas pueden imponer nuevas regulaciones, lo que puede generar la necesidad de refinar las políticas en la práctica constantemente. 

La situación también puede traer una disminución en las oportunidades de trabajo para los nuevos graduados o puede ser difícil para los profesionales existentes hacer frente a las crecientes demandas en las inversiones adicionales ^11^.

## CONCLUSIONES

• Las medidas después de la pandemia del COVID-19 requieren una reflexión para la reanudación de la práctica clínica, especialmente con respecto a los cambios de comportamiento dirigidos a la bioseguridad operativa. Existe un conjunto de alternativas estratégicas y mejoras preventivas específicas que deben planificarse y ejecutarse antes, durante y después de la atención, basadas en la información que surgió durante esta nueva pandemia.
• No existe un protocolo universal para el tratamiento de pacientes con COVID-19.
• El profesional debe estar en constante búsqueda de información actualizada para entregar al paciente un tratamiento humanizado y efectivo, así como para minimizar los riesgos durante el cuidado dental.
• Es importante mencionar que, conforme evolucionan las etapas de la pandemia en los diferentes países, cambian los protocolos de atención; en este sentido, cada país evaluará la evolución de sus protocolos. Por eso, se recomienda al profesional realizar un seguimiento de las actualizaciones propias de su región.

## Fuente de financiamiento:

Autofinanciado
